# Messaging on Slow Impacts: Applying Lessons Learned from Climate Change Communication to Catalyze and Improve Marine Nutrient Communication

**DOI:** 10.3389/fenvs.2021.619606

**Published:** 2021-03-10

**Authors:** Katherine Nicole Canfield, Kate Mulvaney, Nathaniel Merrill

**Affiliations:** Atlantic Coastal Environmental Sciences Division, U.S. Environmental Protection Agency, Office of Research and Development, Center for Environmental Measurement and Modeling Narragansett, RI, United States

**Keywords:** climate change communication, nutrient management, science communication, nutrient communication, science of science communication

## Abstract

Building publics’ understanding about human-environmental causes and impacts of nutrient pollution is difficult due to the diverse sources and, at times, extended timescales of increasing inputs, consequences to ecosystems, and recovery after remediation. Communicating environmental problems with “slow impacts” has long been a challenge for scientists, public health officials, and science communicators, as the time delay for subsequent consequences to become evident dilutes the sense of urgency to act. Fortunately, scientific research and practice in the field of climate change communication has begun to identify best practices to address these challenges. Climate change demonstrates a delay between environmental stressor and impact, and recommended practices for climate change communication illustrate how to explain and motivate action around this complex environmental problem. Climate change communication research provides scientific understanding of how people evaluate risk and scientific information about climate change. We used a qualitative coding approach to review the science communication and climate change communication literature to identify approaches that could be used for nutrients and how they could be applied. Recognizing the differences between climate change and impacts of nutrient pollution, we also explore how environmental problems with delayed impacts demand nuanced strategies for effective communication and public engagement. Applying generalizable approaches to successfully communicate the slow impacts related to nutrient pollution across geographic contexts will help build publics’ understanding and urgency to act on comprehensive management of nutrient pollution, thereby increasing protection of coastal and marine environments.

## INTRODUCTION

There is a large disparity between the scientific and public understanding of the consequences of nutrient pollution. Intentional engagement with localized publics on the significance of the problems created by nutrient pollution and the need for collective behavioral change is essential for achieving management goals. To date, national, regional, and local policies to manage nutrients are a start in translating science for public benefit, but current policies and public engagement do not match the scale of the nutrient pollution challenge. Given the scarcity of this engagement, there is a need for more effective science communication about nutrient pollution and its impacts. We use “nutrient communication” to refer to this needed increase in translation of science and engagement with publics to address nutrient pollution.

Nutrient pollution is increasingly understood in terms of ecological and social impacts, alongside the identification of sources and potential management actions. The primary nutrients of concern are reactive nitrogen and phosphorus. These nutrients occur naturally throughout the biosphere, but the levels of both have been increased significantly through various human activities to the point of polluting our environment. The fact that healthy ecosystems require these nutrients in certain quantities, but they become pollutants at higher levels, makes it difficult to determine and communicate the point at which these nutrients become pollutants (Nixon, 1993; Merrill et al., 2018). Additionally, the combination of point sources, such as concentrated animal feeding operations, and nonpoint sources, such as septic systems and fertilizer run-off from row-crop agriculture, make nutrient pollution difficult to manage. Nonpoint sources are the extensive inputs of nutrients without a single “point” of origin. This nonpoint nature makes nutrients more difficult to manage, as all the diffuse small sources must be managed among many individual actors, often without legal mandate, rather than addressing a singular potent polluting site (EPA, 2020b). The U.S. EPA (2020b) reports that nonpoint source pollution is the main remaining cause of impaired water quality. While addressing nutrient pollution across the range of sources and impaired waterbodies is important, this article focuses specifically on communicating the impacts of nonpoint source nutrient pollution on coastal water quality.

In most coastal waters, availability of reactive nitrogen is most important because it limits primary production more than phosphorus does (Howarth and Marino, 2006). Excess reactive nitrogen can cause heightened algal production and biomass, harmful algal blooms, accelerated coral reef decline (Zaneveld et al., 2016), seagrass loss due to shading, and degradation of fish and other aquatic communities due to low oxygen. Point sources of nutrients include wastewater treatment facilities, stormwater outfalls, and concentrated animal feeding operations (Carpenter et al., 1998). These sources are managed as identifiable “points” of nutrient input, with certain amounts of nutrient inputs permitted. Important nonpoint nitrogen inputs include septic systems (EPA, 1996), agricultural runoff (Van Meter et al., 2016) and atmospheric deposition (Carpenter et al., 1998). An added difficulty in nutrient management is that impacts may occur far downstream from sources, may take an extended period of time to fully manifest, and may persist long after sources have been eliminated (Van Meter et al., 2016; Merrill et al., 2020). Transit time for nutrients from sources to receiving waters can vary from hours to decades, in the latter case usually when transport via groundwater is involved (Van Meter et al., 2018). Additionally, even once reaching a larger waterbody, the impact of the nutrients on the ecology of the system takes time and can be cumulative (Verdonschot et al., 2013). In these cases of delayed impact, the nutrient levels in waterbodies and consequential eutrophication (Pérez-Ruzafa et al., 2019) can reflect nutrient inputs that preceded implementation of nutrient management, delaying recovery of ecosystem functioning (Carstensen et al., 2011). This legacy impact makes it difficult to understand effects of nutrient management interventions and to communicate the importance of such interventions. To address the challenges of aquatic nutrient pollution, research has found that the most effective management plans comprehensively address all nutrient sources (Gross and Hagy, 2017) and integrate multiple scales of decision makers (Greening and Elfring, 2002). Building such management plans requires effective communication to build publics’ awareness about the complexities of aquatic nutrient pollution.

Despite the difficulty (Boesch, 2006) and limited success in building awareness around nutrient pollution (Osmond et al., 2010; Greening et al., 2014; Perry et al., 2020), we argue that the extensive research in communicating climate change can provide insight into effective communication strategies that can motivate public action. Climate change and nutrient pollution have historically progressed slowly, resulting in “shifting baselines” (Pauly 1995) of system status. Originally used to refer to changing fish biomass (Pauly, 1995), shifting baselines for nutrients (Duarte et al., 2009) and climate (Moore et al., 2019) reflect that systems have changed such that the reference baseline level today is different than it was in the past. Additionally, climate change and nutrient drivers are similar in having both major point and nonpoint sources, while impacts are similar in being both localized and widespread. While impacts of nutrient pollution are generally localized, the larger scale of the Haber-Bosch process for industrial production of reactive nitrogen for agricultural use has lowered costs, enabling broader application and thereby expanded the spatial scale of nutrient pollution (Fowler et al., 2013). Higher availability and more widespread use of reactive nitrogen leads predictably to increased losses to surface waters. We argue that the similar challenges in communication for climate change and nutrient pollution of the slow impact, shifting baselines, and diversity of sources create an opportunity for nutrient communication to learn from climate change communication and apply best practices.

**FIGURE 1 |:**
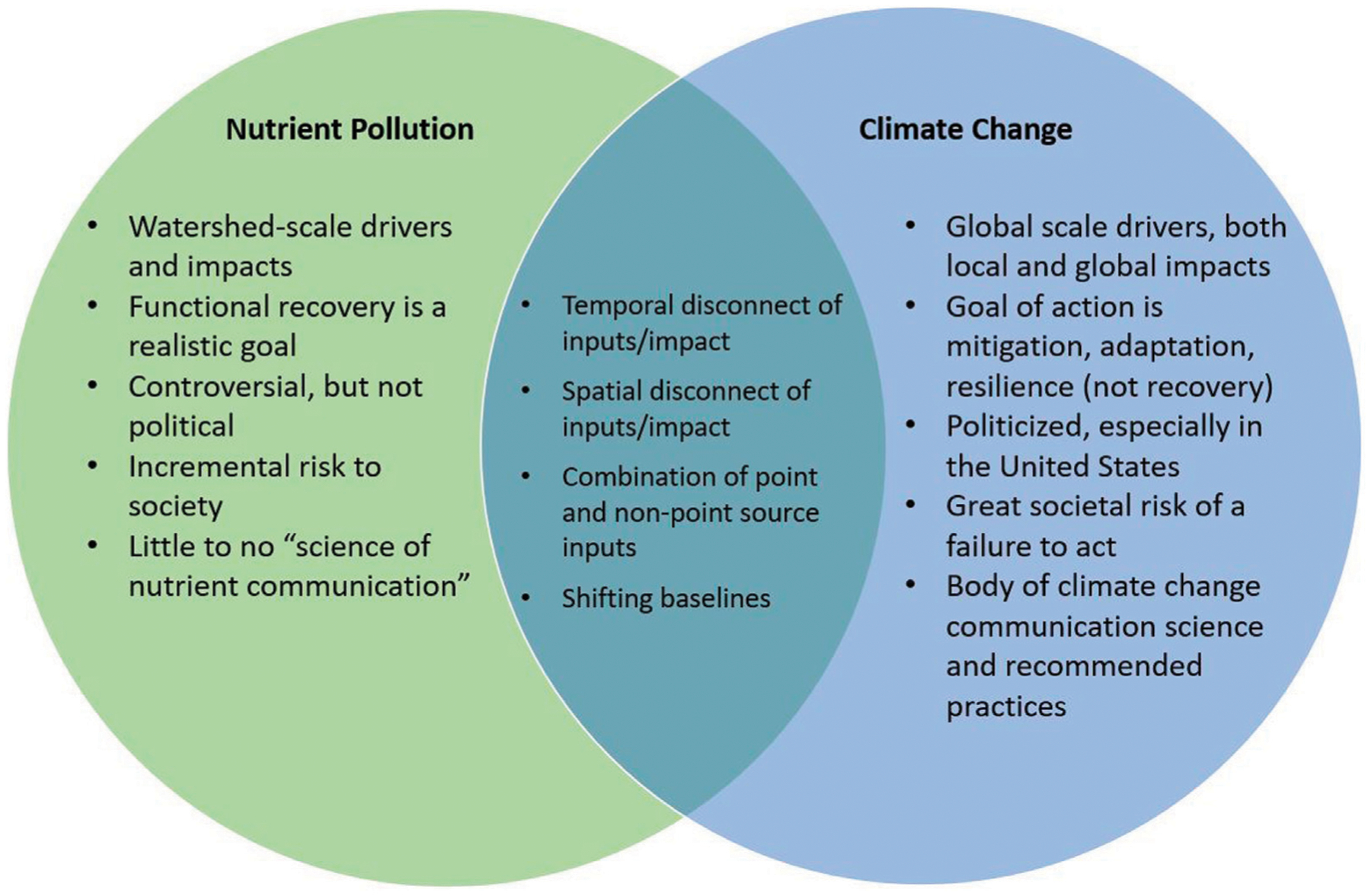
Venn Diagram of similarities and differences between environmental issues of nutrient pollution and climate change.

Along with the many similarities between nutrients and climate change, there are also notable differences. Although both are “slow” the timescale is meaningfully different for the two issues (Figure 1). With nutrients, the entire transition from pristine, to polluted, to recovered could potentially occur within a person’s lifetime (Pérez-Ruzafa et al., 2019). In contrast, many of climate change’s most severe impacts are occurring across generations and the possible time to recovery is unknowable.A further difference is that nutrients generally result in more localized impacts in coastal waterbodies and climate change has less bounded environmental consequences. An additional relevant difference is that there has been extensive interdisciplinary effort to mobilize publics around the globe to act against climate change. We present these examples here to clarify that lessons from climate change are applicable, with tailoring to the nuanced differences of these stressors.

In this paper we respond to the current lack of scientific research on effective science communication on nutrients and management and address the need for researched recommended communication practices. We explain how climate change communication applies lessons of effective science communication within the difficult bounds of motivating action to respond to slow impacts. We then present our analytical approach, which uses qualitatively coding of peer-reviewed and grey literature. In the results section we discuss the findings of our literature review of climate change communication and the ways nutrients science communication may differ from climate change communication in its barriers to effective practice. We then present the best practices drawn from climate change communication that arose as themes from this literature review and integrate applications to nutrient communication in coastal environments. We rely heavily on climate change communication throughout because there is minimal research (Boesch et al., 2001; Boesch, 2006; Osmond et al., 2010; Perry et al., 2020; Reddy et al., 2020) on best practices for nutrient communication. We conclude by reiterating key points about the value of connecting these environmental communication topics that are salient for nutrient scientists and communicators.

### Literature Review

The “science of science communication” refers to the study of the state of science communication and public engagement with science (Fischhoff and Scheufele, 2013). Among other areas of research, past science communication work includes how various social identities are or are not actively included in various science learning environments (Dawson, 2014; Massarani and Merzagora, 2014; Streicher et al., 2014; Canfield et al., 2020), efforts at broadening participation in science (Bevan et al., 2018), ways of knowing in science (Eveland and Cooper, 2013; Medin and Bang, 2014), analyzing how various publics process scientific information and apply it to decisions (Dilling and Lemos, 2011; von Winterfeldt, 2013), assessing the structural limitations to scientists producing useful communications (Anderegg, 2010; Scheufele, 2013), and climate change communication (van der Linden et al., 2015; additional sources below).

As an introduction to the critical approach of the science of science communication, we present potential misconceptions in use of the phrase “public understanding of science.” An initial nuance of communicating science is that rather than there being a monolithic “public” with a shared understanding of science, there are instead a variety of publics. These publics have differing education, experiences, and beliefs that lead to different understandings of science (Kisiel and Anderson, 2010). As an example, the terminology, objectives, and assumptions associated with the knowledge needed to communicate with an audience of elementary school children are different than for a room full of policymakers (Burks and Menezes, 2018).

This conceptualization of “public understanding of science” reveals a rhetorical distance between scientists and non-scientists that complicates building a community or societal understanding of science outside of research institutions. The widely debunked “deficit model” (Wynne, 1992; Nerlich et al., 2010) refers to a one-way transfer of knowledge from experts to lay people, which assumes people who are not in traditional scientific research roles have no scientific understanding, just beliefs based on experiences (Wynne, 1992). This model exemplifies a false dichotomy in scientific understanding. Scholars of the science of science communication point out there are both formal and informal ways people know and are exposed to science (Stocklmayer et al., 2010), including formal education (DeBoer, 2000), informal learning (Reich et al., 2010; Dawson, 2019), and traditional and indigenous ways of knowing (Johnson et al., 2014; Lemus et al., 2014). Differing ways of knowing and beliefs (e.g., political affiliation and religion) result in differential trust in scientific research, especially as related to climate change (Brossard and Scheufele, 2013; Eveland and Cooper, 2013; Wang et al., 2018). Notably, distrust in science also arises based on identity, especially social constructions of race, due to historic and continued exploitation (e.g. HeLa cells and the Tuskegee Study) of people with marginalized identities (Suite et al., 2007; Scharff et al., 2010). How information is accessed and assimilated leads to various communities of understanding and acceptance of science that are the product of more, or less, effective science communication efforts in conversation with historic injustices, social norms, and individual values. With the case of climate change, we present some theories of how these communities of understanding develop, and we describe efforts to conduct science communication that positively impact publics’ understanding.

One of the largest subfields within the field of the science of science communication is specifically focused on climate change communication. This subfield investigates how people process and apply the science of climate change in their daily lives and how communicators can design communications that motivate communal action on climate change. Several failed attempts to communicate and motivate action around climate change have led to extensive research to understand why this specific area of science communication is so difficult. Two important difficulties are 1) conveying the risks associated with a changing climate to people with different ways of thinking and 2) explaining the urgency of action, which we focus on in our results.

## METHODS

In order to identify key themes for communication, we used qualitative coding of peer-reviewed literature. Papers were collated based on the topics of the science of science communication, public engagement with and communication about climate change, and to a lesser degree nutrient science and communication. Papers were identified using Google Scholar, searching keywords and keyword phrases (Mase and Prokopy, 2014). The keyword phrases included “science of science communication,” “climate change communication,” “public engagement with science,” “recommended practices” + “climate change communication,” and “psychology of climate change communication.” Google Scholar was used rather than a specialized academic database due to the interdisciplinarity of the topics of interest and Google Scholar’s ability to support a more inclusive search of relevant scholarly work. Google Scholar has been identified as the most comprehensive academic search engine (Gusenbauer, 2019), and has addressed past concerns over transparency and vetting of articles as the search tool has matured (Halevi et al., 2017; Martín-Martín et al., 2018). When our selected keyword phrases were searched in Web of Science, only climate change communication produced similar results. During review of those papers identified from the initial search, additional relevant papers were identified from the literature cited and were also coded. A keyword search to identify relevant articles on nutrient communication included searching “nutrient communication,” eutrophication + communication, nutrients + communication, and “science communication” + “nutrients,” with only five relevant articles discussing communicating about nutrients (Boesch et al., 2001; Boesch, 2006; Osmond et al., 2010; Perry et al., 2020; Reddy et al., 2020). Articles that took an applied social science approach that presented the state of understanding of the fields and research-informed recommended approaches to science communication, climate change communication, and nutrient communication were included from these searches. Based on the articles identified from keyword searches, a total of 66 articles were coded (see supplementary materials for citations), with additional articles reviewed but not coded when found to be irrelevant.

**FIGURE 2 |:**
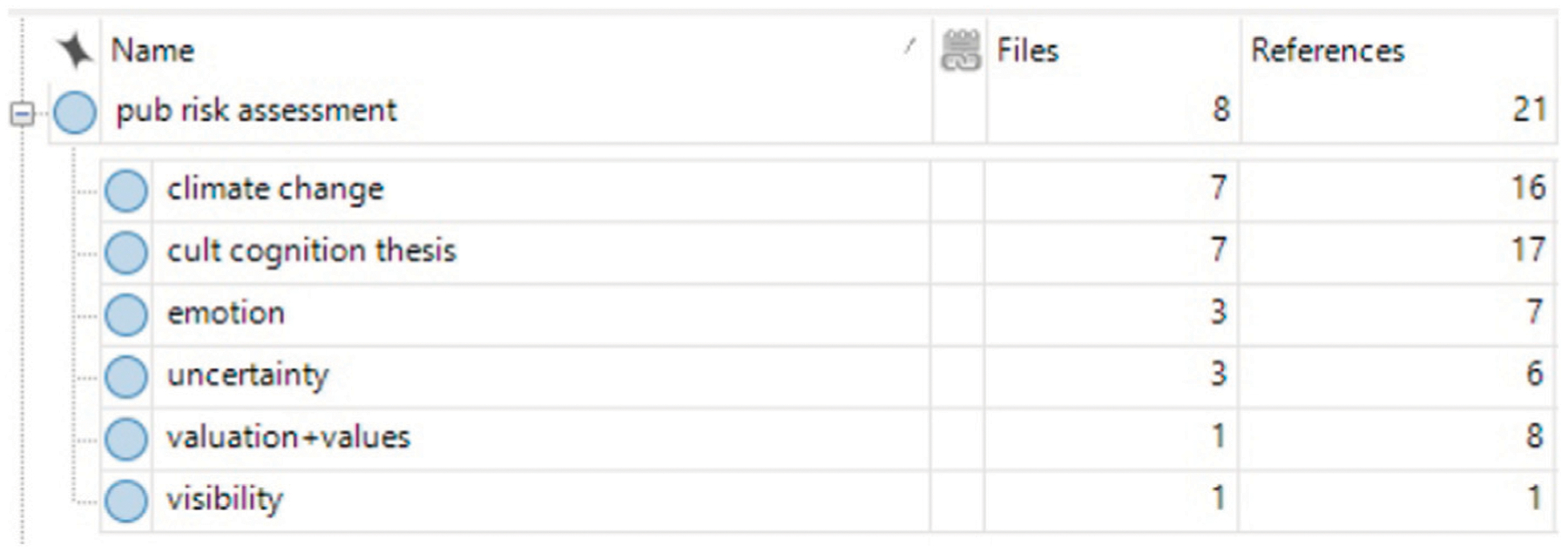
Screenshot of NVivo 12 showing coded articles related to public risk assessment. Public risk assessment is the first tier category, and those listed below it were the codes identified as existing within the larger category.

Qualitative coding is a method that can be applied in multiple ways (Elliott, 2018). The method allows for sifting through dense data such as text or interviews (Creswell, 2015). It can be applied using a systematic approach (Khan et al., 2003), and can also be used to identify emergent themes inductively (Mase and Prokopy, 2014). We used the NVivo 12 qualitative analysis software to inductively identify codes in the selected papers. Emergent themes were identified based on the analytical focus of recommended practices for effectively communicating science, and specifically communicating climate change, leading to nested codes including best practices for science and climate change communication, and academic limitations to effective communication. One researcher was responsible for all coding to ensure reliable and consistent identification and application of codes across papers. A total of 70 codes were identified using a tiered system wherein the first-level category was more general, and within this first tier was a second tier of the related codes that were more pointed or conceptual aspects within the general category (Creswell, 2013; Elliott, 2018). For example, a first tier (relatively general) category was “public risk assessment” in which general comments on public risk assessment were coded. The second-tier codes within this category specify different analyses and topics that affect how publics assess risk as relevant to science: climate change, cultural cognition thesis, emotion, uncertainty, valuation and values, visibility (Figure 2). Recommended practices for climate change communication and nutrient communication were chosen based on the codes that were consistently identified across the literature as best or recommended practice.

## RESULTS

Effective science communication for reducing nutrient pollution is important, but best practices remain greatly understudied, with only five papers found that review nutrient communications. The review of the much broader literature on science and climate change communication therefore provides lessons on the theory and use of science communication for climate change that can then be applied to communicating nutrient pollution. First, we present the findings related to the theory of how people think about climate change and what makes it difficult to convey the urgency to act, followed by explanation of the differences between nutrient and climate change communication. We then describe our thematic findings on the effective practices of climate change communication and their application to nutrients.

### How People Think About Climate Change

Explaining the risks of climate change demands appealing to the different ways people assimilate scientific information through their mental models (Bruine de Bruin and Bostrom, 2013). Mental models refer to how people reconcile scientific information with their beliefs (Einsiedel, 1994; Scheufele, 2013). Mental models are constructed from values that are the sum of lived experiences, education, and beliefs that become tacitly accepted knowledge frames for decision making, risk assessment, and evaluation of scientific information (Fischhoff and Scheufele, 2013). These models have been found to be particularly helpful in predicting people’s behavior in relation to environmental issues, such as climate change, that allow them to dissociate their implication in the problem (Paolisso, 2011). Many people have mental models that allow for accurate interpretation of scientific information (Fischhoff and Scheufele, 2013). These models may have critical gaps in scientific understanding of environmental issues such as climate change, however, due to communication failures such as lack of appeal to emotion and effort to convey complex science (Fischhoff and Scheufele, 2013).

Mental models incorporate two information processing systems: the emotional and the analytical (Slovic et al., 2004; Marx et al., 2007; Roeser, 2012). The emotional system is based on experiences and responds quickly, whereas the analytical system is more deliberate and based on understanding (Marx et al., 2007; van der Linden, 2015). While both of these systems are always used in decision making, some scientists argue that the role analytical processing plays in assessing climate change risk has been overestimated, ignoring the role that emotions play (Slovic et al., 2004; Marx et al., 2007). The role of emotion in risk assessment is known as the “affect heuristic,” or “risk as feeling” (Alhakami and Slovic, 1994; Loewenstein et al., 2001; Leiserowitz, 2006; Roeser, 2012). Slovic et al. (2004) and Marx et al. (2007) argue for increased appeal to risk as feeling, such as personal experience, to address a general underassessment of risk relative to that identified by scientific research. Appealing to emotions provides an alternative to presenting complex climate models and statistics that do not align with people’s existing mental models and may therefore not be accepted or understood (Marx et al., 2007; Fischhoff and Scheufele, 2013). This appeal to the effectiveness of emotion for communication emphasizes that science communication, and specifically (climate) risk communication is not just about accurate science, but the way that science is conveyed to different publics (Hertwig et al., 2004; Weber et al., 2004).

In agriculture, mental models have been used to understand the varied values of farmers and how they make decisions (Eckert and Bell, 2005; Prager and Curfs, 2016). This research helps scholars understanding farming choices (Eckert and Bell, 2005; van Hulst et al., 2020), and can be informative for extension educators (Eckert and Bell, 2005) and policymakers (Prager and Curfs, 2016). To date, this work appears to have not focused on nutrient management or pollution, nor specifically on the communication implications, as reflected by the lack of articles on nutrients and mental models in our literature search.

The cultural cognition thesis supposes that belonging to religious, political, or other social groups can explain the different ways people process information (Kahan et al., 2011; Kahan, 2015) and, like mental models, provides an explanation for how social group membership can impact risk assessment. The argument behind cultural cognition is that people are “cognitive misers” and tend to minimize the amount of thinking they have to do that complicates their existing beliefs, and thus rely on their cultural beliefs to simplify processing of new information (DiMaggio, 1997; Eveland and Cooper, 2013). With this desire to minimize processing of excess information, the thesis explains that people more willingly accept information that aligns with their group affinities rather than considering all information presented as having equal potential to be true. In the case of climate change in the United States, Kahan (2015) found that political affiliation predicts acceptance of climate change as scientific fact better than education level. Cultural cognition explains that belonging to a certain religious (Nisbet and Scheufele, 2009; Kahan, 2015) or political groups (Gauchat, 2012) is associated with amount of trust in science, which affects beliefs about climate change. To avoid overstating the power of this thesis to fully explain multidimensional social issues, we note that political ideology is but one characteristic of an individual, and that work on cultural cognition has been focused largely around the case of differing American views on controversial societal issues (van der Linden, 2016). Thus, we acknowledge that this thesis is a useful example of how group membership impacts interpretation of contentious scientific and societal issues within the U.S. context, but should be carefully applied in other circumstances.

### Conveying Urgency to Act

One climate concept that demands better communication is the urgency of action around climate change (Leiserowitz, 2005; Lorenzoni and Pidgeon, 2006). Compared to the 2014 assessment, the 2018 National Climate Assessment shows increased action among businesses, communities, and governments to reduce the risks of climate change, although current actions were not found to address the full risks of climate change (USGCRP, 2018). The insufficiency of current actions points to the continued gap between the statistical risk of climate change and interpretation of that risk relative to other factors considered by community leaders and decision-makers. Researchers have identified the perceived “remoteness” (Hoijer, 2010) and abstraction of climate change (Leiserowitz, 2005; Spence et al., 2012; Nurmis, 2016; Wang et al., 2018), along with reliance on analytics and statistics (Marx et al., 2007) in communications as causes of such a disconnect between statistical and perceived risk. Whereas the general population does not perceive imminent risk due to climate change, perception of risk increases when the consequences are visible, immediate, and nearby. This difference has been highlighted in 2020, as publics contrasted how the urgency with which the media presented the crisis of, and potential solutions to, the COVID-19 pandemic and the lower urgency associated with climate change, which has made it much less prominent in the major news cycle (Peters, 2020; Regan, 2020; Roth, 2020). Further confusing the perceived sense of urgency around climate change is misinformation on the scientific consensus behind climate change (Cook, 2019), which encourages a dismissal of the threat.

The distancing of oneself from climate change aligns with the understanding that humans tend to prefer immediate over future benefits (Maibach et al., 2008) and, similarly, deferred expenses over immediate sacrifices (Meyer, 2013). Since the benefits of acting on climate change often are at a scale that is difficult for humans to comprehend, there is a lack of motivation to understand the risk or act with urgency. Another potential explanation for the lack of extensive perceived risk of climate change is deemed the “finite pool of worry” (Linville and Fischer, 1991; Madhavan, 2011). As people become more concerned about one given risk, their concern for other risks decreases (Hansen et al., 2004; Marx et al., 2007). For example, when the concerns of Argentinian farmers increased in relation to climate change, their concern about local politics decreased, even though the political dynamics in the community had not changed (Linville and Fischer, 1991). Taken together, abstraction of climate change, a finite pool of worry, and people’s mental models provide a psychological explanation of why climate change risk is rarely acted on, or addressed by, publics at a scale commensurate with the projected impacts.

Social marketing is one approach that has been touted as having great potential to create an urgency to act. Social marketing refers to the systematic use of marketing techniques over the long-term to achieve specific behavioral goals for social good (Lazer and Kelley, 1973). This differs from other kinds of marketing where changing behavior for commercial reasons is the goal (Wiebe, 1952; Maibach et al., 2008). The social marketing approach has become renowned as an effective strategy to go beyond the “pamphlet approach” of providing people information on a subject (Corner and Randall, 2011:1007), and focuses on creating long-term change in specific publics’ behaviors for social good (Fox and Kotler, 1980; Peattie and Peattie, 2009). Building an effective campaign relies on researching consumers values and segmenting the audience of the campaign based on these values to create efforts targeted to different values. A review of ocean sustainability social marketing campaigns found that preliminary research on audience knowledge, identities, and values is essential to achieving the desired campaign outcome and understanding campaign leaders’ choices (Bates, 2010).

Critiques of social marketing as a strategy for climate change communication and engagement point out that these efforts are largely aimed at changing individuals’ behaviors rather than creating community-level, policy, or systemic shifts in practice (Maibach et al., 2008; Corner and Randall, 2011). Additionally, while it has proven advantageous to tailor messaging on behavior change towards the specific intrinsic values of a group (or a specific mental model) (Bolderdijk et al., 2013; van der Linden, 2015), such efforts are not worthwhile if promotion interferes with pursuing the longer-term goal (Corner and Randall, 2011; Corner et al., 2014). In the case of climate change, the larger goal of a societal commitment of addressing fossil fuel emissions requires people to adopt behaviors in line with self-transcendent and pro-environmental values and conservation. However, these goals are incongruent with an audience segment of a social marketing campaign known to have highly materialistic values. Highlighting the monetary benefits of energy efficient light bulbs may appeal to this segment’s self-enhancement values. Ignoring the centrality of environmental sustainability in catering this message, however, will lead to a failure to achieve the larger behavioral change towards conservation-minded and sustainable consumption (Deci et al., 1999). Additionally, one principle of social marketing is the “exchange” of the benefit and cost of behavior change (National Social Marketing Centre, 2006; Corner and Randall, 2011). If the exchange requires an incentive to motivate a behavior change that is contrary to a person’s beliefs, research has found that as soon as the incentive is removed, individuals revert to past practices (Crompton, 2010; Corner and Randall, 2011; Corner et al., 2014). One of the few articles that mentioned effective communication on nutrients noted that incentives need to be associated with education and regulations to create lasting behavior change (Osmond et al., 2010). A final critique notes that communication approaches that “sell” issues to promote public engagement foster caution and cynicism rather than community support (Walls et al., 2005; Doubleday, 2007; Corner and Randall, 2011). Evidence shows that when it comes to publics with pro-environmental values, social marketing promotes positive behavior changes, suggesting how key these values are in behavior change and scientific communication (Maibach et al., 2008; Corner and Randall, 2011). Thus, using social marketing along with other tools from the climate change communication strategy toolbox can help balance the associated benefits and risks.

### Differences Between Climate Change and Nutrient Communication

As demonstrated above, climate change communication has an extensive library of scholarship. Contrarily, five articles were identified as discussing nutrient communication, which presented important lessons learned (Boesch, 2006; Osmond et al., 2010; Boesch, 2019; Perry et al., 2020). Three of these focused on evidence-backed recommendations for communication moving forward (Osmond et al., 2010; Perry et al. 2020; Reddy et al., 2020). Significant space remains for building a more expansive body of literature of evidence-backed practices for nutrient communication. Until then, finding connections to existing bodies of literature can provide valuable support to inform nutrient communication practices.

While there are many similarities in communicating about climate change and nutrient pollution, there are also important differences to be aware of in comparing communication approaches. As already highlighted, the “slowness” of the impacts of climate change and nutrient pollution occur across different timescales. This requires adjusting communications to reflect that climate change impacts are largely intergenerational while nutrient pollution impacts are felt within a generation. Failure to make such adjustment in conveying the impacts of nutrient pollution would inaccurately represent the issue, response rate of the system, and potentially further confuse recipients of such communication. Additional differences we identified were the spatial scale of the environmental challenge, the end goal of publics’ engagement, and the politicization of the challenge in the United States. In addressing these differences, we reiterate the call to adjust the approach as the context changes.

The context of addressing climate change is different than nutrient pollution given the scale of climate change is explicitly global while nutrient pollution impacts are often relatively local in scale. Climate change does have localized impacts, such as coastal flooding from rising sea levels, but these impacts are the result of both local climate change preparedness and global scale management of climate due to the connected nature of the system. Compared to climate change, nutrient pollution results from more localized actions and management (i.e., watershed scale). As a result, its consequences are experienced most directly by humans in the watershed, noting that major rivers can also cross political boundaries and impact downstream users separated from sources, and atmospheric nitrogen pollution is usually regional or national. While those communicating climate change and nutrient pollution need to localize the issue to the scale of the system, the spatial disconnect is often not as extreme for nutrients. This makes localizing the cause and effect for relevant publics more straightforward, as the problem is generally most effectively managed at the local watershed scale (Gross and Hagy, 2017). With climate change, communicators are challenged with identifying relevant local impacts or proxies of a global issue that will be meaningful to the various communities that they work to mobilize (Linville and Fischer, 1991; Marx et al., 2007). As communicators are contending with an issue with both point and nonpoint sources, they must overcome the ease with which people can distance themselves from localized contributions, and the challenge of whose responsibility it is to manage the problem.

Nutrient pollution also differs from climate change in the end goal of public engagement. With climate change, the goal is often to mitigate impacts, adapt to new environments, and build resilient societies rather than to return to a historic environment. In nutrient management, the goal is often to recover the functioning of ecosystems, lakes, or estuaries (Duarte et al., 2009; Verdonschot et al., 2013; Gross and Hagy, 2017). This recovery is often to a different state than the system before becoming polluted (Duarte et al., 2009), but still is a restoration of or return to (Duarte et al., 2015) a functioning system (Carstensen et al., 2011; Pérez-Ruzafa et al., 2019). The full removal of nutrients from a system is not always possible (Palumbi et al., 2008), and past work has called for the need to have realistic goals in nutrient management (Weinstein, 2008). However, the possibility of such restoration of ecosystem functions provides a visually compelling message to motivate publics’ participation in calls for management. Significant improvements in ecosystem functioning are possible within five years of addressing point source pollution (Taylor, 2006), though full recovery in managing larger nonpoint source nutrients takes longer (Lefcheck et al., 2018). While recovery in nutrient pollution cases, such as when nutrient flows have been reduced quickly with sewage treatment plants (Taylor, 2006; Greening et al., 2014), has been observed, rapid shutoff of greenhouse gas emissions to know what recovery from climate change could look like has not been done.

Another difference impacting public engagement is the different severity of risks posed for these two environmental challenges. Nutrient pollution presents important concerns of impaired water quality and in most cases incremental loss of benefits from coastal ecosystems. In contrast, climate change presents impacts that may be extremely severe and have the potential to profoundly change human society. Climate change requires localizing and concretizing an issue that has potential impacts that are yet to be fully realized, whereas eutrophication from nutrient pollution has numerous examples to which communicators can point (Nixon, 1995; Paerl, 1997). The relatively well-defined impacts of nutrient pollution are at a significantly different scale, and are usually less hazardous, compared to the wide-sweeping impacts anticipated from climate change (IPCC, 2014).

Finally, the politicization of climate change in the United States makes communication more difficult than that on nutrients. This necessitates a highly nuanced practice in communicating climate change to people whose political beliefs have become increasingly associated with disbelief in the phenomenon (Anderegg, 2010; Kahan et al., 2012) or those dismissive of critiques of climate science (Van Rensburg and Head, 2017). Nutrients are not free of politicization. During in the 1960s and 1970s the link between phosphates in detergents and water pollution, especially around the Great Lakes was highly politicized. Environmentalists and residents mobilized to call for government action to address water quality. While they were at first at odds with politicians and businesses that claimed detergent companies could self-manage, eventually phosphates were banned from detergents (Kehoe, 1992). While still not an apolitical issue today, nutrient management does not currently face the same national political polarization as climate change and other issues such as genetically modified foods and stem cell research (Kahan, 2015; Kahan et al., 2015). This could be because while there are whole centers focused on identifying how people think about and communicate climate change in the United States (Maibach et al., 2009), centers explicitly focused on understanding how people think about nutrient pollution and science are lacking. While nutrient pollution communicators and scientists may still currently face issues with distrust in science (Bauer, 2006; Scheufele, 2013), they do not have to overcome mass media disproportionally presenting conflicting views (Eveland and Cooper, 2013; Petersen et al., 2019) of the causes and impacts of excess nutrients.

One notable shared difficulty in communicating nutrient pollution and climate change is the lack of clarity in the messaging distinguishing between the overall processes and individual consequences of these challenges. The changing framing within research areas and between disciplines creates different vocabularies to describe issues with the same, or extremely similar sources (see Table 1 for some examples). This creates muddled messages for publics not versed in connecting the processes of climate change or excess nutrients with their consequences. For example, in the literature, *climate change* is consistently used to refer to the societal scale, abstract result of increased greenhouse gas pollution, while some speak specifically about *sea level rise* and others create a distinct discourse about *ocean acidification*. With nutrients, the framing is often *nutrient pollution*, but terminology of *excess nutrients*, or the impacts of *harmful algal blooms* and *eutrophication* are also used to refer to the same problem. When choosing terminology, communicators need to present clear messaging of which terms describe the environmental processes, impacts, and their relationships to improve message effectiveness. Additionally, the framing of the terms eutrophication, algal blooms, climate change, and sea level rise is all based on the impacts of nutrients and greenhouse gas pollution rather than on the sources or inputs. This provides another way for people to distance themselves from their responsibility in contributing to these challenges. Addressing these variations in framing consequences of environmental processes within the community of scientists working on issues related to climate change and nutrient pollution could streamline communication and build collaborative networks of scientists (Anila, 2017). Building a more explicitly defined and agreed upon vocabulary of terms within fields would also make the science more accessible to publics outside of these disciplines, as it would demand scientists clearly define the meanings and bounds of the terms they use.

### Key Themes of Climate Change Communication Practices for Application to Nutrients Communication

The key findings related to practice of climate change communication fit under the themes of the importance of training and the importance of framing. Training refers to preparing scientists and communicators to share their messages or motivate publics. The theme of framing contains topics and analyses on the content and approach for sharing climate change messages with diverse publics. Together, these themes identify both the past shortcomings in climate change communication and recommended approaches for increasing publics’ awareness and action to address climate change.

### Training

Scientific researchers may struggle to produce science communication materials that are useful for their intended audience or users due to a lack of training in, or anticipated reward for, production of such materials (Jacobson et al., 2004; Nisbet and Scheufele, 2009). Some scientists may not know who the relevant or target audience of their work is, due to a belief that science is for knowledge production alone (Dilling and Lemos, 2011) or due to a lack of training and subsequent experience in the identification of relevant users of their science and their needs (Fischhoff and Scheufele, 2013; Nerlich et al., 2010; von Winterfeldt, 2013). An inability to identify end users can result in a tendency to focus communications on what researchers find interesting and important (Bruine de Bruin and Bostrom, 2013; Scheufele, 2013). Not tailoring information for use by publics other than scientists can result in available science being largely comprehensible and accessible for other researchers in a similar research area (Marx et al., 2007; Dilling and Lemos, 2011; Bruine de Bruin and Bostrom, 2013; Scheufele, 2013). Others may want to use science to influence policy, but lack understanding of how to do so (Hetherington and Phillips, 2020). While it is too simplistic to claim that scientists are totally responsible for all scientific communication, the science of science communication emphasizes the need to break down the strict boundaries of categorizing people as scientists or nonscientists in order to produce more useful science communication products.

Lack of training in science communication (Anderegg, 2010; Dilling and Lemos, 2011; Fischhoff and Scheufele, 2013; Scheufele, 2013) highlights whether academic research systems are designed to prioritize effective science communication by researchers (Jacobson et al., 2004), or if that is even a researcher’s role. In the case of universities with extension offices, researchers argue these offices are to serve as information brokers that translate and communicate science to relevant stakeholders (Prokopy et al., 2015). Alternatively, researchers might work with nongovernmental organizations or news media to produce science communication products (Boesch, 2019). However, this still assumes that scientists have the intrinsic motivation, time, and/or skills to work closely with people outside of academia to produce materials for publics outside of their area of expertise. This is not a critique of scientists’ values, but rather a questioning of whether research systems as designed have provided the support for researchers to do science communication beyond academic conferences and papers. The lack of academic rewards for engaging with publics on science (Anderegg, 2010; Dilling and Lemos, 2011; Singh et al., 2014) might explain why researchers may not claim ownership of the task of communicating their science (Dilling and Lemos, 2011). The literature reveals that within the theme of training, there are subthemes, including lack of preparation of scientists in communication, the understanding that scientists are not necessarily science communicators, and a lack of professional recognition for communication work.

### Framing

Framing arose as a theme based on the consistent emphasis across the literature on building messages that are designed for the various ways people assimilate and apply scientific knowledge (Scheufele, 2013). The five topics that emerged as essential for framing are:

1. concrete vs. abstract examples (Marx et al., 2007; van der Linden et al., 2015).
2. mental models (Nerlich et al., 2010; Dilling and Lemos, 2011; Bruine de Bruin and Bostrom, 2013).
3. imagery (Corner et al. 2014; Nerlich and Jaspal, 2014; Metag et al., 2016; Eskjær, 2017; Wang et al., 2018).
4. positive vs. negative messaging (Nerlich et al., 2010; Gifford and Comeau, 2011), and
5. social norms (Corner and Randall, 2011; Gifford and Comeau, 2011; van der Linden et al., 2015; Wang et al., 2018).

Both designing messages with a focus on the concrete rather than abstract and being aware of peoples’ mental models were discussed across the other three topics. Concrete examples based on real weather events (Marx et al., 2007; Bloodhart et al., 2015) and localized experiences (Nicholson-Cole, 2005; Wang et al., 2018; Monroe et al., 2019) have been found to mobilize communities more than relying on abstract ideas or projected models of extreme weather or esoteric statistics (Marx et al., 2007).

Regarding imagery, the literature noted that there is a persistent abstraction in much climate change imagery (Wang et al., 2018). The image of the polar bear, which has become associated with climate change (Doyle, 2007; Leviston et al., 2014; Swim and Bloodhart, 2014), is an abstraction because most humans never interact with a wild polar bear. Other examples of abstractions include use of politicians (Rebich-Hespanha et al., 2014), public figures and protestors (Smith and Joffe, 2009; O’Neill and Smith, 2013), and scientists (Leon and Erviti, 2013). Non-abstract images of climate change could include narratives that outline the impacts of climate change on “ordinary” humans or other stories including humans (Corner et al., 2015) and emotion (Marx et al., 2007; Meldrum et al., 2012) in visualizations. Such visualizations have been found to reduce the psychological distance perceived with climate change (Swim and Bloodhart, 2014; Wang et al., 2018). Specifically, appealing to positive emotions rather than fear has been an important topic in framing climate change messages and imagery (Leviston et al., 2014). Apocalyptic visualizations of climate futures may aim to stand out against the imagery of daily life (O’Neill and Nicholson-Cole, 2009) but instead serve to further distance people from the desired engagement (O’Neill and Nicholson-Cole, 2009; O’Neill and Smith, 2013). As was previously noted, people have a finite capacity for worry at any given time (Linville and Fischer, 1991; Madhavan, 2011). Evidence suggests that to mobilize people around climate change, appealing to motivation is more effective than stoking fear and calling for sacrifice (Nerlich et al., 2010; Gifford and Comeau, 2011).

Activating social norms is another topic relevant to framing and mental models. Activating social norms involves framing climate change as a “social reality” that affects people’s ways of living (Rowson, 2013; Corner and Clarke, 2016; Pearson et al., 2016; Wang et al., 2018). As people are social beings, if family and friends begin to talk about climate change and mobilize to address climate change, individuals will increase their perception of risk and actions to minimize the risk (Renn, 2010; van der Linden, 2014). Rather than trying to frame messages to shift behavior at an individual scale, appealing to social norms activates and leverages community behavior to create larger-scale mobilization to address climate change (Corner and Randall, 2011; Gifford and Comeau, 2011; van der Linden et al., 2015; Wang et al., 2018).

While adjusting climate change frames did not predict behavior regarding a specific farming intervention (Singh et al., 2020), intentional climate change framing effectively increased support for climate policy (Walker et al., 2018). Acknowledging the role of climate change in natural disasters can have negative effects on the processing of scientific facts for climate change skeptics (Dixon et al., 2019), pointing to the importance of considering the mental models the audience in preparing climate change communications. Though these examples do not argue in favor of one specific method of framing, together, these studies exemplify that the actors and audiences to which information is communicated foundationally affect the effectiveness of a message (Reddy et al., 2020). The recommended practices based on these themes that follow emphasize the importance of context in communication.

### Recommended Practices

Our literature review and qualitative coding analysis identified five recommended practices for climate change communication that would also apply to nutrient pollution communication:

1. prioritize two-way communication between publics and communicators,
2. relate to human experience rather than abstract analysis,
3. emphasize local impacts and immediate actions to be taken,
4. define and activate social norms around the problem and urgency of action, and
5. build interdisciplinary collaborations to address science communication training and reward gaps.

Addressing climate change and nutrient pollution with similar communication strategies relies on the similar ease with which publics psychologically distance themselves from their role as causal agents and associated slow and spatially distant impacts. While the principles are transferable, the differences noted above in these challenges necessitate tailoring the principles to the specifics of each stressor and/or situation. Despite the differences between nutrient pollution and climate change, there are similarities in the difficulties of past communication efforts that allow us to learn from scholars of climate change communication. While both the temporal and the spatial disconnect may not be as great for nutrients as with climate change, the shared slow impacts make lessons from climate change communications useful in building motivated publics across sectors to tackle this environmental problem. The five recommended practices for climate change provide an evidence-based starting point to improve communications on nutrient pollution, which we demonstrate with example applications of each of these practices. These examples focus on building publics’ understanding of how nutrients enter and pollute water bodies and actions that communities and individuals can take to reduce nutrient loading.

In all science communication, materials that allow give and take among the audience and those preparing such materials ensures that the right questions are answered (Moser, 2010; Dilling and Lemos, 2011; Corner et al., 2014) and that local knowledge and context is addressed (Collins and Evans, 2007; Nerlich et al., 2010). This first practice for climate change communication aims to ensure the science that is shared is relevant and useful to the intended audience (Bruine de Bruin and Bostrom, 2013; Fischhoff and Scheufele, 2013). The need to prioritize two-way communication builds on the shortcomings and failed efforts documented in the climate change communication literature. Recommended practices 2 through 4 further emphasize the need to include local implications of climate change in communications. These three practices reduce the psychological distance of the response (Swim and Bloodhart, 2014; van der Linden et al., 2015; Wang et al., 2018).

In practice, two-way communication on nutrients can include holding public hearings and other forms of consistent meetings on policy and planned management strategies that allow for public comment, that is then meaningfully incorporated into planning documents. Implementing two-way communication requires recognizing the varied priorities and ways of assimilating scientific information that exist across residents, policymakers, scientists, environmental activists, and other groups of people to ensure communications respond to groups’ values and needs. Further, consistent interaction with people across the various publics via meetings (in-person or virtual) will ensure that management strategies address the needs of local residents and incorporate the historical knowledge residents have of their communities. To be most inclusive, this communication will need to recognize the expertise that comes from lived experiences as well as that from formal education or official status (Ottinger, 2013). Recognizing lived expertise minimizes the risk of a deficit approach of “talking at” (Lewenstein, 2003; Smallman, 2016) or “selling” (Corner and Randall, 2011) nutrient science to publics, so that communicators instead engage in a constructive dialogue (Nisbet and Scheufele, 2009; Smallman, 2016; Monroe et al., 2019).

Explaining the science in relation to ecosystem services and activities that people are familiar with is helpful to reduce the psychological detachment of nutrient management. This is applicable both to recommended practices 2 and 3. Addressing the local slow impacts and spatial disconnect between inputs of nutrients and their impacts requires finding frames for communications that will motivate engagement. Framing the issue considering something important to local identity, such as beach access in coastal towns or the importance of productive farming in agricultural areas, is one transferable tool to concretize the challenge (Mexico Hypoxia Assessment, 2017). Local residents that define themselves based on where they live will most readily accept an appeal to the value of the environment and natural resources to motivate action (Madhavan, 2011).

One example of making nutrient management less abstract is preparation of an infographic that describes the impacts of nutrient pollution on beach and water quality and beach access and closures. In conjunction with an infographic explaining the science and impacts, localizing the actions that can be taken at the individual, town, county, and state scale to mitigate impacts is also important (Greening and Elfring, 2002). Social marketing could be a useful tool in framing the need to build support for these communal actions that protect natural resources for the good of the local economy and environment among those who already display passion for conservation. However, the power of social marketing and message framing to change conservation behaviors should not be overstated. A recent study on nutrient communication strategies found that in the case of farmers not already engaging in conservation behaviors, message framing towards economic and environmental values was less effective at encouraging conservation than just presenting information on the practice (Reddy et al., 2020). Based on the past work on climate change communicators (Marx et al., 2007) and effective nutrient management (Gross and Hagy, 2017), localizing the problem and benefits is essential to community participation.

To create urgency to change behaviors in a lasting way to address nutrient pollution in coastal waters, the relevant social groups that need to be engaged are likely at both the neighborhood and watershed scale. This is in accordance with the literature arguing for mobilizing publics via social norms rather than targeting individuals. These publics include both people who live in these communities and contribute to the nonpoint nutrient loading and those decision makers responsible for the waterbody. Research has found that targeting normative beliefs, that is, what people believe about the behavior of others, is effective for creating behavior change (Maibach et al., 2008; Paolisso, 2011). This suggests that appealing to a community sense of pride in a less impacted environment, regarding nutrients at least, can be effective for mobilizing resident publics. One application of this could consist of informing residents of the severity of water quality impairments to a watershed, and then building campaigns focused on mobilizing the community at different scales to protect the watershed. It is essential to build and appeal to a shared sense of community up to the watershed scale to ensure communal mobilization to address shared problems at a scale that will have a meaningful impact for the impacted waterbody (Boesch, 2006; Merrill et al., 2018). As people within these communities will likely have slightly different mental models, mobilizing around a shared identity will build a sense of connection and responsibility to protect their community. As a past nutrient communication effort found, increasing understanding of the issue alone has not proven effective in overall nutrient reduction; policy and clear actions at multiple scales are needed to encourage actions with urgency (Boesch et al., 2001; Greening and Elfring, 2002; Boesch, 2006; Osmond et al., 2010).

This brings attention to the important point that while behavior change is an important component of nutrient management, it is the responsibility of coordinated efforts across local, regional, and national government agencies to institute plans and policies for nutrient management (Greening and Elfring, 2002). Past work has emphasized the connection between communication and management. One study found that the most common community motivator to call for nutrient management was when publics became aware of an ecological crisis, media attention further increased awareness, and then publics mobilized to demand government action (Gross and Hagy, 2017). For this call to be successful, Gross and Hagy (2017) found that there needed to be a specific ecological goal, such as restoring seagrass habitats (Greening et al., 2014), reconfirming the need for public mobilization around a concrete issue and action. A recent study further emphasized that to address eutrophication effectively requires sustained engagement of various levels of government in concert with publics, as aware constituents can hold officials accountable to meet identified goals (Boesch, 2019).

The final recommended practice for climate change communication that can translate to nutrient communication is the result of the theme of training and the disconnect between research scientists and the public. Changes in training could build partnerships across interested and relevant organizations such that all necessary skillsets are represented. This aims to overcome the reality that no one organization or individual can have training and expertise across all the disciplines or topics, and thus should not be expected to lead in areas in which they have limited or no training. Relevant experts to connect climate scientists with include social scientists, communication scholars (Anderegg, 2010), extension officers (Prokopy et al., 2015), and science communication practitioners (Fischhoff and Scheufele, 2013) or “information brokers” (Dilling and Lemos, 2011). The objective of science communication is to ensure science is accessible and useful for publics. For this to be the case, science communicators have a mediating role of providing clear translation of scientific information to publics, and to ensure scientists understand what scientific questions are of interest to publics. One of the few articles located that discussed communication on nutrient pollution also emphasized the importance of “boundary organizations” that specialize in science communication and can provide necessary support in translating policy and scientific research into useful information that is relevant to community concerns (Boesch, 2006).

The bookend practices of 1 and 5 together emphasize the need to work with individuals across disciplines or official capacities. This is an asset-based approach (Burks and Menezes, 2018) that aims to incorporate into communications the expertise, or assets, of individuals across diverse backgrounds (Banks et al., 2007; Jensen and Holliman, 2015). Taken together, these five practices are evidence-backed ways to improve sharing of information about climate change, nutrient communication, and increasing public engagement. They are practices that science communicators can use to produce useful communications that support and increase publics’ awareness about nutrients and the consequences of pollution, and publics’ understanding of management needs. While management plans require looking at a larger scale of the watershed, communications require relating to specific audiences within that watershed, and humanizing complex science for these audiences. In sum, communications serve to support and advocate for nutrient management via communities’ increased ability to discuss and identify the problem and potential impacts.

## DISCUSSION

The lessons gleaned from the science of climate change communication provide a backbone to improve efforts to communicate about nutrient pollution. A survey at the end of a two-year communication campaign about watershed-scale management of water pollution on Cape Cod, Massachusetts, revealed minimal improvement in communities’ awareness of both the local water quality problem and possible solutions (Perry et al., 2020). This has the potential to be a major problem for this tourism-dependent community, as impaired water quality is associated with reduced recreational value (Merrill et al., 2018). In the Neuse River Basin, North Carolina, agricultural runoff is responsible for over half of the nitrogen loading to the estuary and resulted in algal blooms and fish kills for decades. To address this problem, cooperative extension specialists led a nutrient management training program for farmers that increased awareness of nutrient pollution, which both emphasized having a dialogue and preparing education materials (Monroe et al., 2019). At the conclusion of the training, however, a field survey showed the training did not change farmer practices or nutrient loading to the estuary (Osmond et al., 2010). This exemplifies the need for an approach that goes beyond simply sharing information. The challenges faced in changing behavior in both these efforts show that a strategic communication approach (Besley et al., 2019) is essential, and that communications are not a standalone solution to a systemic problem. Continuing efforts to build publics’ and policymakers’ understanding and buy-in to nutrient management is essential (Druschke, 2013). This must be done in conjunction with management strategies that further encourage and enforce behavior change. Finally, while these efforts leave space for improvement, they also demonstrate a focus on localized impacts and examples that climate change communication could benefit from adopting in concretizing messages.

Throughout this paper, we have strived to show the transferability of communication practices between climate change and nutrient pollution. This has been primarily based on the slow impacts and spatially detached drivers and impacts, but the transferability is also due to the interconnected and widespread nature of these issues. As the climate changes, eutrophication that already impacts most U.S. estuaries (Howarth et al., 2000) is expected to worsen in global waterways (Howarth et al., 2000; Alam and Dutta, 2013). Additionally, improved nutrient management is an important part of mitigating climate change due to gaseous nutrient pollution. When comparing the ability of greenhouse gases to warm the atmosphere, nutrient pollution in air as nitrous oxide is 300x as potent as carbon dioxide (Forster et al., 2007; EPA, 2020a). As these issues are intertwined, communications that encourage behavior and policy that improves environmental quality for one of these issues indirectly benefits the other (Russell et al., 2009). The recommended communications strategies are most definitely applicable in building awareness and presenting behaviors for improved environmental quality for both environmental challenges.

Based on the literature review and qualitative coding of the research, five recommended practices for climate change communication were identified that are easily transferred to nutrient communication. The communication practices we identified share an underlying emphasis on relating communication to the societal and environmental context and recognition of the assets that all relevant publics and individuals have to address the environmental challenge. These practices address the need to communicate intentionally between scientists and communities impacted by nutrient pollution such that communications effectively convey urgency across different audiences. They can be applied in navigating communicating slow impacts in a diversity of settings, including across government agencies. Rather than providing a template, the lessons here are of the transferable communications framing, and the need for a multipronged approach to achieve improvements in environmental quality (Osmond et al., 2010). The recommended approach to nutrient communication demands that communicators localize, don’t catastrophize; continue to learn from existing efforts; and provide action items specific to different publics’ expertise, social groups, and policy power.

As nutrient pollution continues to impact marine waters in the United States and globally and impacts worsen, the need for effective nutrient communication is increasing. The findings from the field of climate change communication provide an important set of evidence-backed practices that can be applied toward improving nutrient communication to mitigate impacts.

## Supplementary Material

Supplement1

## Figures and Tables

**TABLE 1 |:** Different terminology used in framing environmental challenges around either the source or the outcome, with a couple of papers as examples of each. Note that these citations often used more than one of the terms in their issue category.

	Nutrients	Climate
Source	Excess nutrients/nitrogen (Smith et al.(1999), Davidson et al. (2012), Van Meter et al.(2016))	Greenhouse gas emissions (Kennedy et al., 2009; Riahi et al., 2011)
Outcome	Eutrophication (Cloern (2001), Glibert et al. (2013)) Nutrient pollution (National Research Council (2000), Beck and Hagy (2015), Gross and Hagy, (2017)) Harmful algal blooms (Glibert and Pitcher, (2001), Sutula et al. (2017)) Hypoxia/hypoxic zones (Howarth et al. (2011), Van Meter et al. (2016))	Ocean acidification (Doney et al., 2009; Kroeker et al., 2010) Global warming (Root et al. (2003), Zhang et al. (2004)) Climate change (Rosenzweig et al. (2008), Monroe et al. (2019)) Sea level rise (Church and White (2006), Nicholls and Cazenave (2010))
