# Supplementary material for: Messaging on Slow Impacts: Applying Lessons Learned from Climate Change Communication to Catalyze and Improve Marine Nutrient Communication
Source: Front Environ Sci. Author manuscript; Available in PMC 2022 Mar 10. (PMC8040056; doi:10.3389/fenvs.2021.619606)
Supplement: Supplement1 [file NIHMS1683030-supplement-Supplement1.docx]

**Coded Articles by Topic**

**Nutrients Science and Management**

Cha, Y., Stow, C.A. (2015). Mining web-based data to assess public response to environmental events. *Environmental Pollution 198*, 97-99.

Gross, C. and Hagy, J. (2017). Attributes of Successful Actions to Restore Lakes and Estuaries Degraded by Nutrient Pollution. JOURNAL OF ENVIRONMENTAL MANAGEMENT. Elsevier Science Ltd, New York, NY, 187:122-136.

Merrill, N.H. Mulvaney, K.K., Martin, D.M., Chintala, M.M., Berry, W., Gleason, T. R., Balogh, S., Humphries, A.T. (2018): A Resilience Framework for Chronic Exposures: Water Quality and Ecosystem Services in Coastal Social Ecological Systems, *Coastal Management*, DOI:10.1080/08920753.2018.1474066

Van Meter, K.J, Basu, N.B, Veenstra^,^ J.J, and Burras, C L 2016. The nitrogen legacy: emerging evidence of nitrogen accumulation in anthropogenic landscapes. Environmental Research Letters, 11, 035014

Van Meter, K.J., Can Cappellen, P., Basu, N.B. 2018 Legacy nitrogen may prevent achievement of water quality goals in the Gulf of Mexico. *Science*, 360, 427–430.

**Nutrient communication**

Boesch, D.F. (2006). Scientific requirements for ecosystem-based management in the restoration of Chesapeake Bay and coastal Louisiana, Ecological Engineering, 26, 6–26.

Boesch, D.F., Brinsfield, R.B. and Magnien, R.E. (2001), Chesapeake Bay Eutrophication: Scientific Understanding, Ecosystem Restoration, and Challenges for Agriculture. J. Environ. Qual., 30: 303-320. doi:[10.2134/jeq2001.302303x](https://doi.org/10.2134/jeq2001.302303x)

Osmond, D.L., Nadkarni, N.M., Driscoll, C.T., Andrews, E., Gold, A.J., Allred, S.R.B., Berkowitz, A.R., Klemens, M.W., Loecke, T.L., McGarry, M.A., Schwarz, K., Washington, M.L. and Groffman, P.M. (2010), The role of interface organizations in science communication and understanding. Frontiers in Ecology and the Environment, 8: 306-313. doi:[10.1890/090145](https://doi.org/10.1890/090145)

Perry, E.S., Smith, S.N., and Mulvaney K.K. (2020). Designing solutions for clean water on Cape Cod: Engaging communities to improve decision making. *Ocean and Coastal Management*, <https://doi.org/10.1016/J.ocecoaman.2019.104998>

**Science of Science Communication**

Brossard D. and Scheufele D.A. 2013. Science, New Media, and the Public. *Science,* (6115), 40-41. DOI: 10.1126/science.1232329

Brossard, D., Lewenstein, B., Bonney, R. (2005). Scientific knowledge and attitude change: The impact of a citizen science project, *International Journal of Science Education*, 27:9, 1099-1121, DOI: [10.1080/09500690500069483](https://doi.org/10.1080/09500690500069483)

Eveland WP Jr., Cooper KE. (2013) An Integrated Model of Communication Influence on Beliefs. Proc Natl Acad Sci USA **110**:14088–14095

Bruine de Bruin W, Bostrom A. (2013). Assessing what to address in science communication. *Proceedings of the National Academy of Sciences USA*. 2013;110 Suppl 3(Suppl 3):14062-14068. doi:10.1073/pnas.1212729110

Fischhoff B. 2013. The sciences of science communication. *Proceedings of the National Academy of Sciences USA*. 110 Suppl 3(Suppl 3):14033-14039. doi:10.1073/pnas.1213273110

Fischhoff B, Scheufele DA. 2013. The science of science communication. Introduction. *Proceedings of the National Academy of Sciences USA.*110 Suppl 3(Suppl 3):14031-14032. doi:10.1073/pnas.1312080110

Kahan, D. M., Jenkins-Smith, H., and Braman, D. (2011). Cultural cognition of scientific consensus, Journal of Risk Research, 14:2, 147-174, DOI: [10.1080/13669877.2010.511246](https://doi.org/10.1080/13669877.2010.511246)

Kahan, D.M. (2015). ‘What is the “science of science communication”?’. Journal of Science Communication 14(03), Y04: 1-12.

Linville, P.W., Fischer, G.W. (1991). Preferences for separating and combining events: a social application of prospect theory and the mental accounting model. *Journal of Personality and Social Psychology Bulletin, 60*, 5–23.

Loewenstein, G., Weber, E.U., Hsee, C.K., Welch, N. 2001. Risk as feelings. *Psychological Bulletin 127* (2), 267–286.

Nisbet, M.C. and Scheufele, D.A. (2009). What's next for science communication? Promising directions and lingering distractions*. American Journal of Botany, 96*, 1767-1778. doi:[10.3732/ajb.0900041](https://doi.org/10.3732/ajb.0900041)

Paolisso, M. Nov 2011. “Why Culture Is Important to Environmental Behavior” in *Integrating the Social Sciences into Chesapeake Bay Restoration* prepared by Chesapeake Bay Program’s Scientific and Technical Advisory Committee.

Peattie, Ken & Peattie, Sue, 2009. "Social marketing: A pathway to consumption reduction?," Journal of Business Research, Elsevier, vol. 62(2), pages 260-268.

Scheufele, D. A. Aug 2013. Communicating science in social settings. *Proceedings of the National Academy of Sciences, 110* (Supplement 3) 14040-14047. DOI: 10.1073/pnas.1213275110

**Climate Change Communication**

Anderegg, W.R.L. The Ivory Lighthouse: communicating climate change more effectively. *Climatic Change* **101,**655–662 (2010). https://doi.org/10.1007/s10584-010-9929-z

Bloodhart, B., Maibach, E., Myers, T., & Zhao, X. (2015). Local Climate Experts: The Influence of Local TV Weather Information on Climate Change Perceptions. *PloS One*, 10(11), e0141526. <https://doi.org/10.1371/journal.pone.0141526>

Corner A, Randall, A. (2011). Selling climate change? The limitations of social marketing as a strategy for climate change public engagement. *Global Environmental Change*, 21:1005–1014.

Corner, A., Markowitz, E. and Pidgeon, N. (2014), Public engagement with climate change: the role of human values. WIREs Clim Change, 5: 411-422. doi:[10.1002/wcc.269](https://doi.org/10.1002/wcc.269)

Corner, A., Webster, R., & Teriete, C. (2015). Climate visuals: seven principles for visual climate change communication. Oxford: Climate Outreach.

Dilling, L., & Lemos, M.C. (2011). Creating usable science: Opportunities and constraints for climate knowledge use and their implications for science policy. Global Environmental Change-human and Policy Dimensions, 21, 680-689.

Dixon, G., Bullock, O. & Adams, D. (2019) Unintended Effects of Emphasizing the Role of Climate Change in Recent Natural Disasters, Environmental Communication, 13:2, 135-143, DOI: [10.1080/17524032.2018.1546202](https://doi.org/10.1080/17524032.2018.1546202)

Doyle, J. (2007). Picturing the Clima(c)tic: Greenpeace and the Representational Politics of Climate Change Communication. Science as Culture, 16(2), 129–150. <https://doi.org/10.1080/09505430701368938>

Gifford, R. & Comeau, L. (2011). Message framing influences perceived climate change competence, engagement, and behavioral intentions. *Global Environmental Change. 21.* 1301-1307. 10.1016/j.gloenvcha.2011.06.004.

Hansen, J., Marx, S., Weber, E.U. (2004). The Role of climate perceptions, expectations, and forecasts in farmer decision making: the Argentine Pampas and South Florida. IRI Technical Report 04-01. International Research Institute for Climate Prediction, Palisades, NY.

Hoijer, B. (2010). Emotional anchoring and objectification in the media reporting on climate change. *Public Understanding of Science , 19(*6), 717–731. <https://doi.org/10.1177/0963662509348863>

Kahan, D. M., Jenkins-Smith, H., Tarantola, T., Silva, C. L. and Braman, D. (2015). Geoengineering and Climate Change Polarization: Testing a Two-Channel Model of Science Communication. *Annals of the American Academy of Political and Social Science 658*, pp. 192–222. DOI: 10.1177/0002716214559002.

Kahan, D. M., Peters, E., Wittlin, M., Slovic, P., Ouellette, L. L., Braman, D. and Mandel, G. (2012). The polarizing impact of science literacy and numeracy on perceived climate change risks. *Nature Climate Change* *2*, 732–735. DOI: 10.1038/nclimate1547.

Leiserowitz A. (2006). Climate change risk perception and policy preferences: The role of affect, imagery, and values. Climatic Change, 77:45–72.

Leiserowitz, A. (2005). American risk perceptions: Is climate change dangerous? Risk Analysis, 25, 1433–1442.

Leon, B., & Erviti, M. C. (2013). Science in pictures: Visual representation of climate change in Spain’s television news. *Public Understanding of Science, 24*(2), 183–199. https://doi.org/10.1177/0963662513500196

Leviston, Z., Price, J., & Bishop, B. (2014). Imagining climate change: The role of implicit associations and affective psychological distancing in climate change responses: Implicit associations with climate change*. European Journal of Social Psychology, 44*(5), 441–454. <https://doi.org/10.1002/ejsp.2050>

Lorenzoni, I., Nicholson-Cole, S. and Whitmarsh, L. (2007). Barriers Perceived to Engaging with Climate Change among the UK Public and Their Policy Implications. *Global Environmental Change*, *17*, 445-459. <http://dx.doi.org/10.1016/j.gloenvcha.2007.01.004>

Lorenzoni, I., Pidgeon, N.F. 2006. Public views on climate change: European and USA. Perspectives Climatic Change, 77: 73–95.

Maibach, E.W., Roser-Renouf, C., Leiserowitz, A. 2008. Communication and Marketing As Climate Change–Intervention Assets, American Journal of Preventive Medicine, Volume 35(5), 488-500.

Marx, S.M., Weber, E.U. Orlove, B.S., Leiserowitz, A., Krantz, D.H., Roncoli, C., Phillips, J. 2007. Communication and mental processes: Experiential and analytic processing of uncertain climate information, Global Environmental Change, 17(1),47-58.

Meldrum, H., Szymanski, D. W., Oches, E. A., Davis, P. T. (2016). A Picture is Worth a Thousand Words: Commentary of Broadcast Meteorologists on the Visual Presentation of Climate Change. International Journal of Social Ecology and Sustainable Development, 7 (4), 1-16.

Metag, J., Schäfer, M. S., Füchslin, T., Barsuhn, T., & Kleinen-von Königslöw, K. (2016). Perceptions of Climate Change Imagery: Evoked Salience and Self-Efficacy in Germany, Switzerland, and Austria. *Science Communication, 38*(2), 197–227. <https://doi.org/10.1177/1075547016635181>

Nerlich, B., & Jaspal, R. (2014). Images of Extreme Weather: Symbolising Human Responses to Climate Change. *Science as Culture, 23*(2), 253–276. https://doi.org/10.1080/09505431.2013.846311

Nerlich, B., Koteyko, N. and Brown, B. (2010), Theory and language of climate change communication. *WIREs Climate Change, 1,* 97-110. doi:[10.1002/wcc.2](https://doi.org/10.1002/wcc.2)

Nicholson-Cole, S. A. (2005). Representing climate change futures: a critique on the use of images for visual communication. Computers, Environment and Urban Systems, 29(3), 255– 273. <https://doi.org/10.1016/j.compenvurbsys.2004.05.002>

Nurmis, J. (2016). Visual climate change art 2005-2015: discourse and practice. Wiley Interdisciplinary Reviews. Climate Change, 7(4), 501–516. <https://doi.org/10.1002/wcc.400>

O’Neill, S. J., & Smith, N. (2013). Climate change and visual imagery. Wiley Interdisciplinary 31 Reviews. Climate Change, 5(1), 73–87. <https://doi.org/10.1002/wcc.249>

Monroe, M.C., Plate, R.R., Oxarart, A., Bowers, A., & Chaves, W.A. (2019) Identifying effective climate change education strategies: a systematic review of the research. *Environmental Education Research, 25*(6), 791-812, DOI: [10.1080/13504622.2017.1360842](https://doi.org/10.1080/13504622.2017.1360842)

Prokopy, L.S., Carlton, J.S., Arbuckle, J.G. Haigh T, Lemos MC, Saylor Mase A*.,* Babin, N., Dunn, M. Andresen, J., Angel, J., Hart, C., Power, R. (2015). Extension′s role in disseminating information about climate change to agricultural stakeholders in the United States. *Climatic Change* **130,**261–272. <https://doi.org/10.1007/s10584-015-1339-9>

Rebich-Hespanha, S., Rice, R. E., Montello, D. R., Retzloff, S., Tien, S., & Hespanha, J. P. (2014). Image Themes and Frames in US Print News Stories about Climate Change. *Environmental Communication*, 9(4), 491–519. <https://doi.org/10.1080/17524032.2014.983534>

Renn, O. (2010). The social amplification / attenuation of risk framework: Application to climate change. *WIREs Climate Change, 2,* 154–169.

Roeser, S. (2012). Risk communication, public engagement, and climate change: A role for emotions. Risk Analysis, 32(6), 1033–1040. [https://doi.org/10.1111/j.1539-6924.2012.01812.x](https://psycnet.apa.org/doi/10.1111/j.1539-6924.2012.01812.x)

Singh, A.S., Church, S.P., Dang, L., Hennes, E.P., Prokopy, L.S. (2020)*.* Does climate change framing matter? Evidence from an experiment of crop advisors in the Midwestern United States. *Climatic Change*. <https://doi.org/10.1007/s10584-020-02703-8>

Smith, N. W., & Joffe, H. (2009). Climate change in the British press: the role of the visual. *Journal of Risk Research, 12*(5), 647–663. <https://doi.org/10.1080/13669870802586512>

Spence, A., Poortinga, W., & Pidgeon, N. F. (2012). The psychological distance of climate change. *Risk Analysis, 32*, 957–972.

Swim, J. K., & Bloodhart, B. (2014). Portraying the Perils to Polar Bears: The Role of Empathic and Objective Perspective-taking Toward Animals in Climate Change Communication. *Environmental Communication, 9*(4), 446–468.

van der Linden S., Maibach E., and Leiserowitz A. (Nov 2015). Improving Public Engagement With Climate Change: Five "Best Practice" Insights From Psychological Science. *Perspectives on Psychological Science, 10*(6),758-63. doi: 10.1177/1745691615598516

van der Linden, S. (2015). Intrinsic motivation and pro-environmental behaviour. *Nature Climate Change, 5,* 612–613.

van der Linden, S. L. (2014). The social-psychological determinants of climate change risk perceptions: Towards a comprehensive model. *Journal of Environmental Psychology, 41, 112–124*

Walker BJA, Kurz T, Russel D (2018) Towards an understanding of when non-climate frames can generate public support for climate change policy. *Environmental Behavior, 50*, 781-806. <https://doi.org/10.1177/0013916517713299>

Wang, S, Corner, A, Chapman, D, Markowitz, E. (2018). Public engagement with climate imagery in a changing digital landscape. *WIREs Climate Change*. 9:e509. <https://doi.org/10.1002/wcc.509>
